# Mental Health Risk Factors and Parole Decisions: Does Inmate Mental Health Status Affect Who Gets Released

**DOI:** 10.3390/ijerph16162950

**Published:** 2019-08-16

**Authors:** Kimberly A. Houser, E. Rely Vîlcică, Christine A. Saum, Matthew L. Hiller

**Affiliations:** 1 Department of Law and Justice Studies, Rowan University, Glassboro, NJ 08028, USA; 2 Department of Criminal Justice, Temple University, Philadelphia, PA 19122, USA

**Keywords:** prison release, parole decision making, mental health, United States

## Abstract

Parole decision—the decision to release an incarcerated individual from prison conditionally—is one of the most critical decisions across justice systems around the world. The decision carries with it significant consequences: for the freedom of the individual awaiting release (the parolee); for the safety of the community in which they will return; and for the correctional system overall, especially its organizational capacity. The current study attempts to add to the parole decision-making literature by specifically analyzing the role that mental health factors may play in explaining parole decisions. Research to date is inconclusive on whether or not mental illness is a risk factor for criminal behavior; despite this, individuals with mental health problems generally fare worse on risk assessment tools employed in justice decisions. The study relies on a 1000+ representative sample of parole-eligible individuals in Pennsylvania, United States. To increase reliability, the analyses test for several mental health factors based on information from different sources (i.e., self-reported mental health history; risk assessment tool employed by the Parole Board; and risk assessment tool employed by the Department of Corrections). To address validity concerns, the study controls for other potential correlates of parole decisions. Although the multivariate models explained a considerable amount of variance in parole decisions, the inclusion of mental health variables added relatively little to model fit. The results provide insights into an understudied area of justice decision making, suggesting that despite the stigmatization of mental illness among criminal justice populations, parole board members in Pennsylvania, United States, appear to follow official guidelines rather than to consider more subjective notions that poor mental health should negate parole release.

## 1. Introduction

The mentally ill are among society’s most marginalized [1,2,3]. Corrigan and Watson [4] (2002) argue that many individuals with serious mental illness are challenged doubly—“On one hand, they struggle with the symptoms and disabilities that result from the disease. On the other, they are challenged by the stereotypes and prejudice that result from misconceptions about mental illness” (p, 16). As a result, the mentally ill are more likely to be unemployed [5], have limited access to adequate healthcare [4], live in poverty [6], and be homeless [1,4,7]. It is perhaps, therefore, unsurprising that there is a disproportionately high percentage of mentally ill in the criminal justice system [3,8,9]. For the mentally ill who find themselves in prisons and jails, the perceived stigma of mental illness within these institutions often results in their unwillingness to disclose their condition, seek treatment or be compliant with medication [1,9,10], further worsening their clinical condition.

Although some studies suggest modest support for a link between mental illness and violence [11,12], many scholars argue that mental health status is not an independent contributor to violent behavior [13,14]. This is evidenced when we consider that the majority (51%) of state prisoners with a mental health disorder are incarcerated for nonviolent crimes [9]; yet, correctional administrators suggest that they are one of the most difficult populations to manage [15]. As such, they are more likely to be charged with an infraction, i.e., violation of a formal rule or regulation by an inmate in the custody of the Department of Corrections [16,17], serve longer periods of incarceration [9], and return to the criminal justice system [9,18]. Despite this, Skeem, Winter, Kennealy, Louden, and Tatar [19] (2014) suggest that while there is empirical support of a direct association between psychiatric symptoms and a “small but important minority of offenses” (p. 222), the relationship between psychiatric disorders and criminal risk/violence is largely indirect. Using a matched sample of parolees to compare typical risk factors (e.g., antisocial patterns, unstable family/marital) with factors unique to mental illness (e.g., clinical symptoms), Skeem et al. [19] (2014) found parolees with mental illness shared many of the same criminogenic risk factors as nonmentally ill parolees; however, they differed substantially in their degrees of risk for recidivism. Overall, their findings suggest that the mentally ill are more likely to reoffend due to general criminogenic risk factors than symptom-based offending.

The stereotype that mentally ill are prone to violent and criminal behavior is, however, deeply rooted in public opinion [4,14,20]. Studies suggest that the media has long cultivated and reinforced this stereotype [21,22,23,24]. Indeed, Parrott and Parrott’s [23] (2015) review of U.S. fictional crime-based television programs found that the mentally ill were disproportionately portrayed as violent and criminal. This is, they argue, the “initial step in stigmatization, informing attitudes and subsequent prejudicial behavior in the real world” (p. 641). Unfortunately, the stigmatization and stereotyping of mental illness is not, however, “confined to the uninformed public, but includes trained professional from most mental health disciplines” ([4], p. 16).

It is against this backdrop that we explore the role of mental health in explaining parole decision making drawing on a large representative sample of parole-eligible candidates in a large United States’ (U.S.) State. More specifically, we tested for both direct and indirect effects of mental health factors on parole decision making—granting or denial of early release—drawing from two actuarial risk assessment instruments, as well as self-report mental health status, while controlling for criminal risk factors typically considered in parole deliberations (e.g., antisocial attitude, unstable family, unemployment) for their possible mediating role. The results provide insight into a largely unexamined area of parole decision making. We begin by reviewing the pathway for many mentally ill into the criminal justice system and their adjustment to the prison environment. We then discuss the current state of knowledge on parole decision making and then more specifically the role of mental health on parole decision making.

### 1.1. The Pathway to the Criminal Justice System for the Mentally Ill

In the mid-20th century, support for state funded psychiatric facilities in the U.S. began to wane; pharmaceutical companies were discovering antipsychotic medications (e.g., Chlorpromazine, Reserpine), touting that patients could now lead independent lives in the community, and civil rights activists were calling for reform of civil commitment standards culminating into what is termed the deinstitutionalization movement [3,25]. Although arguably well intentioned, the deinstitutionalization movement was a “complex and multifaceted phenomenon” resulting in the release of large numbers of mentally ill persons from psychiatric facilities into the community ([25], p. 177); however, with failed investment in community health care services and diminishing funds for mental health care, many of the mentally ill found themselves living on the streets with deteriorating clinical conditions visible to both the public and the police [3,25].

The move from the institution to the community meant that police were now being thrust into the role of “triage service” providers to the mentally ill and serving as “primary gatekeepers” for both the criminal justice and mental health care systems ([26], p. 23). Indeed, in a study of U.S. police encounters with mentally ill individuals, Bonovitz and Bonovitz [27] (1981) found mental illness-related encounters with police increased by 227% from 1975 to 1979. Thus, it is not surprising that the percentage of mentally ill in prisons and jails also increased.

Rather than moving the mentally ill back to the community setting, many were finding themselves in correctional facilities—the term trans-institutionalization was now replacing deinstitutionalization [28]. Today, more than a third of prisoners (37%) and almost half of jail inmates (44%) in the U.S. have a mental health disorder, with 1 in 7 prisoners and 1 in 4 jail inmates meeting the threshold for a serious psychological disorder (the number of state prisoners with a serious psychological disorder is three times that of adults in the standardized U.S. general population; for jail inmates, five times higher than that of adults in the standardized U.S. general population [29]). Research has determined that “Los Angeles County Jail, Chicago’s Cook County Jail, and New York’s Riker’s Island Jail each hold more mentally ill inmates than any remaining psychiatric hospital in the United States” ([30], p. 1). Greater than three times more individuals with serious psychological impairments enter prisons or jails than hospitals in the U.S. [31]. Accordingly, Torrey [32] (1995) refers to prisons and jails as America’s new mental hospitals. Deinstitutionalization is, however, not unique to the U.S. with many European countries also experiencing the exponential growth of mentally ill in criminal justice facilities [33].

### 1.2. Mentally Ill in the Prison System

The needs of the mentally ill in prison are many and immediate, making adjustment to the institution difficult [3]. Despite their high treatment needs, only about one-third (34%) of state prisoners and 17% of jail inmates in the U.S. will receive treatment, which is most commonly being prescribed medication rather than talk therapy [9]. Moreover, the structure and stressors of the prison environment (e.g., loss of autonomy, overcrowded and noisy conditions, rigid discipline) are especially difficult for seriously mentally ill inmates, often displayed through worsening symptomatic behaviors, diminishing clinical conditions [3,32,34,35,36,37], and “irrational opposition to the demands placed on them” ([38], p. 3).

For the seriously mentally ill, “undeveloped or underdeveloped coping mechanisms” may impair rational decision making to meet the demands of the institutional environment ([38], p. 8). Indeed, there is strong empirical support for the association between psychiatric disorders and disciplinary infractions (see [9,16,17,35,36,39,40,41,42,43,44,45]). Using a large sample of female state inmates, Houser and Welsh [17] (2014) found women with mental health disorders were more than twice as likely to be charged with institutional misconduct compared to women with no disorders. Adams [40] (1986) further suggests that mentally ill inmates are more likely to engage in patterns of misconduct reflective of their clinical conditions (e.g., self-segregation, setting fires to their cells, self-injurious behavior, lack of hygiene, destroying state property). More recently, a survey of mental health directors of state and federal correctional facilities reported that inmates most likely to self-injure are those with psychiatric diagnoses [46]. Ironically, these symptomatic responses to the stressors of the prison environment are often considered a violation of inmate codes of conduct and result in charges or sanctions and subsequent longer incarceration stays.

Correctional officers often lack adequate training to distinguish intentional misbehaviors from clinical manifestations of disorders (see [3,16,36,42,45]). Few states offer more than four hours of correctional officer training on how to respond to mentally ill prisoners [3]. Indeed, inmates with mental health disorders are more likely to report serious disciplinary actions taken for their infractions [16] and are disproportionately represented in segregation units [37,38,47,48,49]. The isolation effect of segregation may worsen their clinical condition (see [50] for a full discussion) and may preclude participation in treatment programs [48,51]. Houser et al. [16] (2012) suggest that for many mentally ill, the use of disciplinary sanctions in lieu of needed treatment service may serve to worsen behaviors, leading to additional infractions and longer stays of incarceration.

The implications of disciplinary charges are many (e.g., restricted housing, loss of privileges), including denial of early release [52]. Indeed, studies have shown that the mentally ill spend longer periods of time in prison and jail than their nonmentally ill counterparts [9,31]. This raises the question of whether clinical manifestations of disorders through maladjustment to the prison environment reduce the likelihood of receiving early release through parole for mentally ill inmates.

### 1.3. General Research on Parole Decision Making

Much research on parole decision making has focused on the factors that impact the conclusions of the parole board members. Parole decisions are complicated because the primary goal is to grant release only to those individuals who will not recidivate and continue to be a threat to their community. Prediction of future actions, especially criminal behavior, however, is not an exact science. Risk assessment and actuarial tools can be helpful in determining which inmates are the best candidates for parole and have gained an acceptance in criminal justice system decision making [53]. Indeed, we know that a multitude of factors, both prior to and during the period of incarceration, come into play that may impact decision makers in the discretionary parole process. These include both static factors (individual attributes that are unchangeable, such as demographics or prior criminal history, which cannot be improved over time), and dynamic factors (attributes that are amenable to change over time and whose influence on one’s behavior can be mitigated in time, such as drug involvement). Moreover, some researchers have argued that parole board members’ decisions are more often based on personal intuition than structured guidelines [54]. Indeed, it has been argued that the main factor in parole decision making is the structure of the parole board rather than the relative qualities of the inmate’s case [55]. Thus, it is apparent that factors other than criminal risk are considered and that parole board members may be using considerable discretion in their release decisions. As a result, it is questionable as to whether these decisions are made in the best interests of public safety.

Traditionally, the inmate’s institutional behavior is one of the main elements the parole board examines when reviewing an inmate’s suitability for release. It is logical that trouble following rules and poor conduct while incarcerated may be related to these behaviors after release, negating the likelihood of success on supervision or desisting from future offending. Accordingly, previous research has found that sentences have been modified on the basis of the inmate’s actions while in prison (see [56] for a review of empirical research, [57]). Interestingly, researchers who conducted interviews with inmates up for parole found that institutional behavior counts only when it is adverse behavior, including noncompliance with treatment programs, and that this relates to parole denial; completion of treatment programs and good institutional behavior were not sufficient reasons to grant parole [54]. Moreover, Vîlcică [58] (2018) examined factors that impacted parole decision making and identified as the two most robust predictors of the granting or denying of parole the indicator of noncompliance with institutional programming (which decreased the odds of being granted release by 97%) and the indicator of institutional misconduct (which decreased the odds of parole release by 91%).

Criminal history and current crime severity are, perhaps not surprisingly, primary factors examined with regard to parole decision making. For example, Turpin-Petrosino [59] (1999) examined several factors that might influence parole outcomes and found that the type of crime for which an inmate was incarcerated was the most important factor. Further, the researcher found that inmates who had committed the most serious and violent offenses were more likely to be denied parole than were inmates who were incarcerated for less serious crimes. Vîlcică [58] (2018), too, examined the role of the nature of the original offense in explaining parole decisions, among a host of factors tapping into punishment satisfaction. She found that the type of offense predicts who gets released, while the amount of time served does not. The researcher suggests that the parole board is “averse to granting release to inmates serving sentences for offenses generally regarded as heinous crimes, regardless of anything else that may help assess those inmates’ readiness for reentry into society (e.g., successful institutional programming and behavior). If a person is in prison for a sex offense, there seems to be little that he or she can do to improve the likelihood of gaining release (p. 1372)”.

The amount of time an inmate has been incarcerated is another area studied by researchers on parole decision making. Carroll and colleagues [60] (1982) found that 80% of Pennsylvania inmates were released at their hearing because parole members thought that having served at least the minimum amount of time satisfied the judge’s sentence. Caplan [56] (2007) looked at the interaction between time served and crime severity and found that punitive considerations are stronger in jurisdictions where there is no minimum time required prior to parole eligibility. Carroll and Burke [61] (1990) found that in Wisconsin, a state with no minimum time, parole board members place greater weight on punishment-relevant issues, such as crime seriousness and prior record, when deciding parole release. Vîlcică’s [58] (2018) examination of inmates eligible for parole found that time served was not related to parole decisions at the multivariate level, though she did find that inmates must have experienced at least one parole denial in order to increase their chances of release, suggesting that parole decision makers use the parole process as a punitive means.

Much research has been conducted on the impact of parole members’ own views on their release decisions which are inherent to the discretionary nature of parole. For example, opinions of the commensurability between crime and punishment have been found to relate to how board members correct for too lenient or too severe sentences [59]. One study of federally sentenced women in Canada suggests that it is the parole board’s assessment of the offender’s ability to change positively that is a main factor in granting parole. The researchers further argued that the parole board placed a high value on women taking responsibility for their past behaviors, overall finding that dynamic risk factors (amenable to change) were more important than static ones (those not amenable to change or intervention) [62]. Taken together, this research supports the idea that parole boards may be using their decisions as punitive measures.

Parole decisions may also be influenced by input provided to the board from the crime victims; this typically entails victims submitting an impact statement and participating in the parole hearings. It has been found that victim participation was a highly significant predictor of parole decision making: When the victim submitted impactful evidence against release, the prisoner was less likely to be granted parole [63]. Another study, however, found that victim input, both positive and negative, was not a significant predictor of parole release, but measures of institutional behavior, crime severity, and criminal history were significant. It should be noted, however, that verbal input had more of an impact than did written input [64].

Although victim input into parole has become routine across many jurisdictions, there is relatively little empirical research on this important issue. In fact, Roberts [65] (2009) examined studies related to victim impact statements and concluded that victims’ opinions regarding release may be a threat to the integrity of the parole decision. For example, her review cited qualitative evidence indicating that parole decision makers view the presence of the victim as a factor undermining their ability to be objective in their assessment of parole candidates’ risk [66]. Similarly, a survey of parole boards across the U.S. found that the number of victims had a stronger influence on their decisions to release than the offenders’ behavior in prison; moreover, research also reports that input from the victim seems to carry more weight than input from law enforcement officials involved in the inmate’s case [67].

### 1.4. Mental Health and Parole Decision Making

With regard to mental health and its impact on parole, the research is limited, and findings have been inconsistent; some studies found inmates with mental health problems are less likely to be paroled than nonmentally ill inmates [68,69], while others that mental health issues do not appear to impact parole decision making [70,71]. Caplan [56] (2007) conducted a review of the existing literature and found mental health status to have a negative impact on parole decisions and also to be one of the main influential factors on parole decisions. This assertion is backed by Feder’s [68] (1994) research that found inmates with a past involving psychiatric hospitalization were 30 times less likely to be released for parole compared to those who never had a psychiatric hospitalization, even controlling for race, prison infractions, and the violence of the current offense.

As we have seen, there are a variety of static and dynamic factors that may impact parole decisions; offender risk, which encompasses both types of factors (e.g., history of violence and pro-criminal attitudes), is one of the most significant considerations. Matejkowski and his colleagues [71] (2011) examined whether criminal risk factors that are part of the parole board members’ decision-making process intervened in the relationship between mental health and parole release. Findings at the bivariate level showed that the percentage of inmates with mental illness released to parole (44%) was not significantly different from the percentage of inmates without a mental health disorder who received a favorable parole release decision (51%). Moreover, the researchers were not able to find any moderating effects of the risk factors on mental illness and parole release in the multivariate analyses. They determined that mental illness was associated with substance use disorder, antisocial personality disorder, and violent charges while incarcerated; however, these factors were not directly or indirectly related to release decisions.

There has been some research that has examined gender with regard to mental health and parole decision making. Hannah-Moffat [69] (2004) studied parole data for a sample of women (*n* = 144) in a federal prison in Canada and found that having been diagnosed with a mental disorder and being perceived as a disciplinary problem were significantly correlated with a negative parole decision. The researcher found that a rejection of release was related to the woman’s mental health and her potential for violence. One illustration of this was a case where the evidence of a woman’s self-harm was viewed by the parole member as an indication of possible violence towards others. Hannah-Moffat and Yule [62] (2011) examined a small sample of violent women offenders (*n* = 59) and assessed whether mental health factors related to parole release decisions. Interestingly, the researchers determined that while being diagnosed with a mental health disorder or having a psychologist who was supportive of parole did not relate to these decisions, having a psychiatric report on file was related to the likelihood of being paroled.

Parole decision making and subsequent parole or denial of parole have important consequences for offenders as well as on their communities and larger society. If those affected by mental health issues never receive parole as a result of these discretionary decisions, it is likely these offenders will max out of their sentence, leaving prison with no supervision. This is a policy concern, since studies have found that parole supervision can aid those with mental health problems in making a successful transition into the community [72]. Thus, a denial of parole, whether justified or not, is likely to play a critical role on the reentry and recidivism outcomes of these offenders, which in turn has a significant impact on public safety.

### 1.5. Parole Decision-Making Process

**Figure 1:**
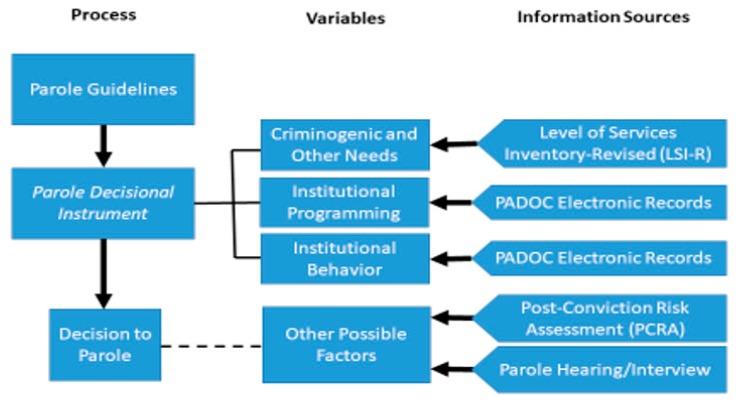
Heuristic of the Pennsylvania parole decision-making process.

A heuristic of the parole decision-making process in the jurisdiction under study is presented in Figure 1. As shown, the Pennsylvania Board of Probation and Parole (PBPP) relies on parole guidelines to inform its decision-making processes. As a part of this, a parole decisional instrument is scored, which summarizes information relevant to the board’s evaluation of parole candidates. Three key domains are emphasized, including reoffending risk, institutional programming, and institutional behavior. An interview was performed with each inmate by a PBPP hearing officer. The interview records, among others, the parole candidate’s self-assessed mental health history. Additionally, the PBPP conducted an official assessment of risk for reoffending using the Level of Service Inventory-Revised (LSI-R) [73], a well-known, validated actuarial risk tool widely used in U.S. corrections and in many other western countries [74]. It is a 54-item interview that measures both criminogenic and non-criminogenic needs. Criminogenic need areas assessed included criminal history, educational achievement, employment history, family and marital status and function, use of leisure time, antisocial associates, drug and alcohol abuse, and antisocial attitudes and behaviors. Non-criminogenic needs included mental health history and status.

A PBPP officer also aggregated information from the Pennsylvania Department of Corrections (PADOC) management information systems that documented participation in institutional programming (e.g., substance abuse treatment, cognitive behavioral therapy), as well as compliance with or unwillingness to participate in required programs. Similarly, PADOC records were used to inform the parole board about individuals’ institutional behavior, which was recorded as a composite of five measures of serious prison misconduct, all occurring within the last year since their last review. Additional information from the PADOC includes individuals’ visitation record while in prison.

One other information source, the Post-Conviction Risk Assessment (PCRA) is used by the PADOC to establish an individuals’ risk for recidivism. The PCRA is a “scientifically based instrument developed by the Administrative Office of the U.S. Courts to improve the effectiveness and efficiency of post-conviction supervision.” ([75], p. 1). Although the PCRA was not routinely shared with the PBPP during the time the current data were collected, it is possible that the parole board did review this information for some parole candidates. The dashed line in Figure 1, therefore, conveys that the PCRA was not commonly available, but it nevertheless is included in the current study because its content overlaps considerably with the LSI-R. Of primary focus to the current study are the components of the PCRA that established mental health history and function. The specific parts of both the LSI-R and the PCRA used as the source for predictors in our models are described more fully below in the Methods section of the paper.

## 2. Methods

### 2.1. Research Setting

Pennsylvania is a discretionary parole state where parole represents the main means for release from prison for nondeath penalty and nonlife sentences. A person becomes eligible for release consideration once they have served the minimum sentence; thus, inmates are usually scheduled for their parole hearings as the expiration of their minimum approaches. Inmates from all PA state correctional institutions (*N* = 26) were included in the sample.

### 2.2. Sample

A random sample of 15% of parole decisions (*n* = 1253) made by PBPP in January–April 2008 was selected for study. It included both parole and re-parole decisions, all involving candidates seeking to gain release after having served their minimum sentences, making them eligible for a parole board review. Of this sample, a total of 81 cases were excluded from analysis because individuals were paroled to detainers (*n* = 75), which did not involve any decision by the parole board, and because decisions were still pending (*n* = 6) when data were collected. The final analytic sample (*n* = 1172) evidenced limited amounts of missing data. Across variables, between 1% and 7% of data were missing, and analysis showed they were missing completely at random; therefore, imputation of missing data was not performed.

Demographically, as shown in Table 1, the largest proportion of the inmates was male (92%) and non-White (58%), and the mean age was 36 years. In terms of nature of the offense controlling the inmates’ sentences, about 40% of the sample were serving sentences for crimes against persons, 18% for property crimes, 25% for drug offenses, and the remaining 17% for other types of offenses. As for institutional custody level, similar proportions of inmates were classified as “minimum” (39%) or “medium” (36%) security, with the remaining classified as “close” or “disciplinary”. Lastly, regarding the sentence being served, the average maximum term was 95 months (almost 8 years); the average minimum term was 33 months (2.75 years); and the average amount of time served by the time of the parole hearing was 48 months (4 years) (Table 1 presents the sample characteristics).

### 2.3. Measures

Dependent variable. The outcome variable, the parole decision, is a dichotomous measure, coded 1 for parole release and 0 for parole denial. With respect to this, about 58% of the inmates were granted parole and 42% were denied. This is consistent with the overall trend in parole decisions during other time periods.

Mental health status. Information related to candidates’ mental health status was derived from three specific sources. One is the information on candidates’ mental health status as reported by eligible inmates to parole hearing officers. To distinguish this from other mental health measures, this is designated as the “self-reported” measure in our analyses. The self-reported mental health history item, a binary variable, was coded 1 when a history of mental health illness was noted and 0 when there was no such indication. The LSI-R was the second source of information regarding inmates’ mental health. Here, five specific items comprising the “Emotional and Personal” subscale in LSI-R, were deemed particularly relevant. More specifically, these items were whether emotional issues created moderate interference in one’s life (Question # 46); whether emotional issues created severe interference or indicated active psychosis (Question #47); whether the inmate had undergone mental health treatment in the past (Question #48); whether the inmate is undergoing mental health treatment currently (at assessment time) (Question #49); and lastly, whether or not there is an indication of a psychological assessment (Question #50). In the current study, we tested each as an individual variable (0 = No, 1 = Yes) rather than as a composite score representing the average of the five items.

The final source of mental health information was the PCRA. Here, specific items were again selected based on their relevance to this study’s focus on mental health-related factors. More specifically, we included whether there was an indication of anger management problems (PCRA Risk #10); indication of history of impulsivity (PCRA Risk #16); indication of suicide attempts (PCRA Risk #22); indication of hallucinations, paranoid delusions, and/or violent fantasies (PCRA Risk #23); and lastly, indication of history of noncompliance to treatment and medication (PCRA Risk #24). All were binary measures (0 = No, 1 = Yes).

Control variables. To increase confidence in the validity of any effects that might be observed for mental health variables, the study controlled for a host of other potentially relevant variables. First, the analyses accounted for the key factors of the parole the Parole Decisional Instrument. These include the LSI-R overall risk score, the overall institutional programming score, and the overall institutional misconduct score. Additional variables reflecting institutional programming included whether an individual completed (0 = No, 1= Yes) any programing, as well as whether violence prevention programming was attended (0 = No, 1= Yes) while in prison. Variables reflecting institutional misconduct included whether any infraction was committed in the preceding year (0 = No, 1= Yes) and whether the infraction involved a threat, blackmail or extortion (0 = No, 1= Yes).

Additional factors expected to influence parole determinations, and thus included for statistical control purposes, included whether the Department of Corrections warden/superintendents’ recommended parole (0 = No, 1= Yes), prior convictions (0 = No, 1= Yes), the nature of the offense controlling inmates’ sentences, and institutional custody levels (see [57,59,61,63,64,76,77]). This study accounted for the following controlling offense-type measures: drug offenses, property, rape/sexual assault, and robbery offenses. Each of these was a dichotomously scored variable (0 = No, 1 = Yes). For example, if the controlling offense was robbery, the robbery variable would have been scored a 1, but all others would be scored a 0 on this measure. Property offenses comprised burglary, theft, and other offenses against property, while drug offenses included mostly manufacturing/drug distribution, possession of drugs, and other related offenses. In identifying the correlates of parole decisions in the analyses screening stage, we also employed an overall violent crime measure that comprised other personal offenses in addition to rape/sexual offense and robbery (such as assault and kidnapping). The results indicated that the overall measure was not significantly associated with the likelihood of parole release; however, the bivariate results identified significant associations, and in different directions, for robbery and rape/sex offense, respectively. Thus, to be able to capture these distinctive associations and to avoid multicollinearity issues, in subsequent multivariate stages, we employed only the individual items for robbery and rape/sex offense and dropped the overall violence measure. Regarding institutional security classification levels, an orthogonal set of dummy variables were used to contrast the medium and close security levels with the minimum security. In addition to these, prior criminal history also has been shown to predict parole decisions [57,60,61,64,77,78,79]. In this study, we relied on a general measure of prior convictions (measure often employed in studies of recidivism). Following other parole decision-making research [56,63,64,70,76,77], the study also controlled for time served (length of incarceration from commitment to parole interview date). More recent research [58,80] also indicates that prior parole reviews and visitation of inmates while in prison may also influence the likelihood of a candidate’s gaining release; thus, the current analyses also controlled for these two factors (binary measures for both). Lastly, the pool of control variables also included sociodemographic factors (race, gender, age, and marital, employment, and education status at prison admission). Only those that were statistically significant in bivariate correlations with parole decisions in this sample were retained for control purposes. In short, the analyses aimed to control for a variety of control variables in efforts to identify any unique influence that mental health factors may have on parole decisions. Table 1 also presents the descriptive statistics for all variables included in the final multivariate models.

### 2.4. Analytic Strategy

The ultimate aim of the analyses was to determine whether mental health factors uniquely predicted subsequent parole release net the effects of other factors explaining variation in parole decisions. In a preliminary step, we employed an approach recommended by Hosmer and Lemeshow [81] (2002) where variables were screened against the parole hearing outcome at a relaxed significance level (*p* < 0.25). As noted by Hosmer and Lemeshow [82] (2013), this relaxed significance level permitted our carrying statistically nonsignificant variables at the bivariate analysis stage into the multivariate analyses. This was done because it would also allow for detection of possible interaction effects between statistically significant and nonsignificant variables and possibly clarify relationships among variables. We used crosstabs (Chi-Square tests) for categorical variables and simple logistic regression (SLR) for continuous variables.

Those variables that passed this initial screening stage were used at the multivariate stage—after being screened for multicollinearity as well. In the final analytic step, we ran a series of multiple logistic regression analyses predicting whether parole was granted (1 = Yes, 0 = No). The first model included control variables but not mental health variables. Four subsequent models, including the different sets of the mental health variables (based on the data source and heuristic model, see above) were then estimated for comparison to the control variable only model. Models 2, 3, and 4 included the self-reported mental health, the LSI-R mental health variables, and the PCRA mental health variables, respectively. The final model includes all mental health variables simultaneously.

## 3. Results

### 3.1. Bivariate Analyses

The results of the bivariate analyses showed that most of the control and the mental health variables were significantly related to parole decisions (See Table 2). The variables from the Parole Decisional Instrument, reflecting the parole guidelines, were the most strongly related to these decisions. Findings showed that higher LSI-R scores were related to a lower chance of being paroled. Having been noncompliant in any programing was related to a much lower chance of being granted parole; those completing a program successfully had a much higher chance for being granted for parole. Serious institutional misconduct in the past year or since one’s last parole hearing was related to a significantly lower chance of parole.

A number of other control variables, consistent with the literature, were associated with parole decisions, with the magnitude of most of these being smaller than the parole guideline variables. For example, having a history of escape or walk-off attempts in the preceding five years was weakly associated with parole recommendation. Having other prior convictions, a controlling offense for robbery, a property crime, or a drug crime were weakly associated with a greater likelihood of being paroled. Three variables, having been recommended for parole by the warden/superintendent, having a controlling offense of rape or sexual assault, and being visited in prison, showed moderately strong relationships with the parole board’s decisions.

All but two of the mental health measures, self-reported mental health and the LSI-R item reflecting severe interference or psychosis, were statistically significantly related to parole decisions at the bivariate level. The magnitude of the associations was generally small, similar to those seen for the other control variables, with the PCRA items indicating anger management problems and a history of impulsivity the strongest of these. Interpretations of these results were all the same; the presence of psychological problems was associated with a smaller chance of being paroled. For example, 53% of those individuals having psychological problems that moderately interfered with their functioning (LSI-R item) gained release versus 63% of those not having such problems (*Chi-Square* = 8.39, *p* < 0.01). Likewise, having hallucinations or delusions, as reported on the PCRA, decreased chances one would be paroled (29% of those individuals were released v. 59% of candidates not reporting such problems; *Chi-Square* = 11.73, *p* < 0.001).

### 3.2. Multivariate Analyses

Having found associations at the bivariate level, the next stage was to determine whether these persisted in the presence of other covariates and the extent to which they improved efforts to explain variation in parole decisions. As noted above, a total of 5 models were calculated, with the first including control variables only as predictors. Subsequent models included different sets of the mental health variables, with the final model including all control and all mental health variables. To summarize, as shown in Table 3, the values for B coefficients, odds ratios (OR), and significance levels for the control variables remained nearly identical across all models. These models explained a considerable amount of variance in parole decisions, but mental health variables added comparatively little to model fit.

Control model. Several factors emerged as robust predictors of the granting or denying of parole in multivariate modeling (see Model 1 in Table 3). Not surprisingly, the predictors from the key areas from the Parole Decisional Instrument (i.e., recidivism risk, institutional programming, and institutional behavior), which aggregated information from interviews with the parolee and from official records in relation to Parole Guidelines, had the greatest apparent influence on parole decisions. With respect to recidivism risk, the OR of 0.97 (*p* = 0.005) for total LSI-R score showed that for every 1-point increase in this, the likelihood of being paroled fell by 3%. Results for institutional programming showed that having been noncompliant with programs was strongly related to a lower likelihood (OR = 0.03, *p* = 0.000) of being paroled; however, completing a program tripled (OR = 3.17, *p* = 0.000) and participating in antiviolence programming nearly tripled (OR = 2.83 *p* = 0.000) the odds of being paroled. Any serious institutional misconduct in the year preceding the hearing decreased the odds of being paroled by 92%, and if the misconduct involved threatening, blackmailing or extorting an individual, it decreased the odds by nearly two-thirds (OR = 0.39, *p* = 0.001). The DOC recommendation, too, carried great weight. A positive recommendation almost tripled (OR = 2.63, *p* = 0.001) the likelihood of parole release. Having prior convictions increased the likelihood of being paroled, but a controlling offense of rape or sexual assault significantly decreased chances for parole (OR = 0.29; *p* = 0.001). However, a controlling offense for robbery, property or drug offense increased the likelihood of parole. Recent escape attempts reduced chances for parole by one-third (OR = 0.61, *p* = 0.001). Having been visited while in prison increased the likelihood of parole (OR = 0.67, *p* = 0.001), but being over age 35 at the parole hearing (OR = 0.63, *p* = 0.030) or being in close (i.e., maximum) security (OR = 0.53, *p* = 0.033) decreased the likelihood of being paroled.

Mental health models. Models 2 through 5 add the primary variables of interest (i.e., mental health) to the control model. Findings from Model 2 showed self-reported mental health problems were not uniquely associated with the probability of being paroled. Likewise, Model 3 indicates none of the LSI-R mental health variables were uniquely, significantly related to the likelihood of parole. Model 4 shows one significant, unique impact of the PCRA mental health variables, with a history of impulsivity (OR = 0.61, *p* = 0.037), a finding that is repeated in Model 5 when all mental health variables are included simultaneously in the model. Two additional control variables emerged in Model 5 as significant predictors. Here, medium custody status (OR = 0.60, *p* = 0.028) was related to lower likelihood and a prior parole hearing (OR = 1.64, *p* = 0.038) with higher likelihood of being paroled. Comparison of the explanatory power of the control model with that of the predictive models including all mental health measures showed a nominal change in model fit, as evidenced by similar *Pseudo-R Square* and *−2 Log Likelihood* for Models 1 and 5. 

## 4. Discussion

Decision making takes place at every step of the criminal justice system, with discretion being an inevitable part of the decision-making process [83]. Parole boards have a great deal of discretion with their decision making and are influenced by a host of criteria [83], which as Hanser [84] (2010) notes, may not necessarily be reflected by statute or official agency guidelines. Parole boards are essentially tasked with predicting future behavior—the likelihood that an offender will reoffend—and the ability of offenders to be responsive to rehabilitation and community supervision [60,83,84]. The implications of parole board decision making cannot be understated, as they are responsible for balancing the safety of the larger community with individual freedom.

With the disproportionate representation of mentally ill in prison, concern about the economic and social consequence of this growing population has led to a vast wealth of research. However, our understanding of mental health status as an independent contributor to parole decision making is both limited and mixed (see [71] 2011 for a more thorough review of the empirical evidence). To provide more insight into this largely unexamined area of parole decision making, we examined specific mental health indicators for their unique contribution in parole decision making net the effects of other factors explaining variation in parole decisions.

Overall, our findings suggest that in this United States jurisdiction, specific mental health indicators are not directly related to parole decision making. Our findings are similar to those of a study conducted by Matejowski, et al. [71] (2011) in a different United States jurisdiction, which also found that mental illness did not have a direct effect on release decisions. Although our multivariate models explained a considerable amount of variance in parole decisions, adding mental health variables to the models added comparatively little to model fit. Impulsivity was the only significant mental health variable—inmates with histories of impulsivity were more likely to be denied parole (OR = 0.61, *p* = 0.037). It should be noted that this measure is included in the PCRA used by the PADOC and is not routinely shared with the PBPP; however, it is possible that the parole board did review this information for some parole candidates.

Several control variables remained significant after mental health indicators were entered into the regression equations (see Table 3, Model 5). As expected, program noncompliance and serious institutional misconduct reduced the likelihood of being paroled. These are key areas from the Parole Decisional Instrument. Moreover, inmates receiving favorable recommendations from the DOC were significantly more likely to be granted parole. Interestingly, inmates with prior convictions were more likely to be granted parole (OR = 1.69). Age was also significantly related to parole decision making, with older inmates more likely to be denied parole, as well as those in serving time in close (i.e., maximum) security facilities.

Having found little support for the independent contribution of mental health status on parole decision making, our findings suggest that despite the stigmatization of mental illness, parole board members in Pennsylvania, United States, appear to more closely follow official guidelines rather than to consider more subjective notions that poor mental health should negate parole release. This being said, although mental health status may not have had a direct influence on parole decision making, it is possible that there was an indirect effect—as the mentally ill are more likely to have risk factors unique to mental illness, as well as significantly more general risk factors for recidivism than their nonmentally ill counterparts [19]. Therefore, it is unsurprising that the mentally ill tend to perform poorly on criminal justice risk assessments [60,69]. It follows then that if the parole board is basing decisions primarily on criminal risk, then mentally ill inmates would be less likely to be paroled.

As part of a comparison study of parolees with and without mental illness on recidivism, Skeem et al. [19] (2014) assessed parolees using the “Central Eight” general risk factors for recidivism—the risk assessment section of The Level of Service/Case Management Inventory (LS/CMI) [73]. Their findings showed mentally ill offenders scored higher on several factors—antisocial patterns, family/marital, education/employment, and pro-criminal attitude orientation. Interestingly, all of these risk factors are considered by the parole board in Pennsylvania as part of the Level of Service Inventory-Revised, a risk assessment tool that is widely used in the U.S. and Canadian criminal justice systems. Thus, although parole boards should be commended for their objective use of evidence-based risk assessment in their decision making, the mentally ill may be indirectly discriminated in parole decision making by virtue of their high rates of general risk factors. For example, impulsivity, which we found to be significantly related to parole decision making, is associated with disorders such as adult attention-deficit/hyperactivity disorder (ADHD) [85] and borderline personality disorders [86]. Persons with these disorders tend to suffer a myriad of symptoms and consequences of those symptoms, such as those found on criminal justice risk assessment tools (e.g., unstable relationships, substance abuse; poor employment history).

The mentally ill are also more likely to have difficulty adjusting to the prison environment; as such, they tend to be charged with more institutional misconduct than nonmentally ill inmates [16,17] and are more likely to be placed in solitary confinement, which has been found to exacerbate their symptoms [87]. Houser and Belenko [42] (2015) contend that punitive responses to the seriously mentally ill serves to worsen their behavior and essentially “sets them up” for further charges of misconduct. The results of our study found institutional misconduct to be a robust predictor in parole decision making—any serious institutional misconduct in the year preceding a parole hearing reduced the likelihood of being paroled by 92%.

In addition, we found that program noncompliance significantly reduces the likelihood of parole. Although U.S. courts have established the constitutional right of prisoners to receive medical care that meets minimum standards (*Ruiz v. Estelle*, 1980), including the right to mental health treatment (*Bowring v. Godwin*, 1977), surveyed estimates of mental health treatment in correctional settings show only 34% of state prisoners and 24% of federal prisons receive treatment [9]. Correctional institutions have a difficult time meeting the treatment needs of the large number of mentally ill [3], leaving many to receive psychotropic medication in lieu of traditional therapy programs [88,89]. Moreover, mentally ill inmates in segregated housing typically lose access to treatment services, receiving instead medication and brief mental health checks [48,51]. Limited exposure to programs further reduces the opportunity to earn good-time credits toward early release [90].

For many mentally ill, their disorders are exacerbated by the co-occurrence of a substance use disorder (COD), requiring integrated treatment programming to address both disorders simultaneously [16,17,91]. However, correctional treatment services are often fragmented, offering either substance use treatment or mental health treatment, thereby excluding this population from needed treatment services [37]. Research across multiple countries and correctional agencies has been consistent in demonstrating that a primary method to reduce prison misconduct *and* recidivism is through effective correctional programming [92,93,94,95,96]).

### Limitations and Future Research

Study limitations should also be noted. In particular, the research relied on the use of certain measures as proxy indicators of mental health status. Arguably, these may be valid indicators; however, inferences from the analyses conducted here would be stronger if qualitative data were also available. Observations of parole hearings and interviews with parole board members regarding their deliberation process and criteria would be especially illuminating. Future inquiries into parole decision making should attempt to add such qualitative analyses. For now, we note that, as most decision-making studies, this is an inferential one. Relatedly, the archival nature of agency data is known to present limitations; however, they are widely employed in studies of justice decisions. Lastly, although Pennsylvania is a large U.S. jurisdiction, which serves a very diverse population and relies on a parole process similar to that employed in other discretionary prison release jurisdictions, we cannot claim generalizability of this study’s findings to other jurisdictions in the United States or elsewhere.

All this being acknowledged, we also note that the study draws on a rich dataset, is based on a large sample, and employed rigorous analytic techniques. The key dependent variable involved no measurement assumptions and the study employed multiple measures for the mental health status as the main independent variable of interest. A host of control variables was also employed. In sum, all of this should offer confidence in the reliability and validity of the study’s results.

## 5. Conclusions

The findings of our study suggest that intervention for criminal justice involving mentally ill people should begin at the time of their admission. Knowing that the mentally ill have a higher propensity to criminogenic risks and needs than their non-mentally ill counterparts and are less likely to successfully adjust to the prison environment, greater attention and resources should be allocated to screening, assessment, and treatment to meet the needs of this burgeoning offender population. Correctional officers need additional training to more accurately assess and differentiate intentional misbehaviors from symptom-based behaviors to more efficiently respond to the needs of the mentally ill rather than respond punitively. Serious mental illness may impair rational decision making and the ability to comply with institutional and program structures. If we increase mental health-specific programs and better train staff in general programs to work with mentally ill inmates, we can improve program compliance and completion and perhaps reduce official misconduct.

## Figures and Tables

**Table 1:** Sample (*n* = 1172).

Sample Characteristic	Count	% or Mean
Gender		
Male	1079	92.1%
Female	93	7.9%
Race ^a^		
Black	537	45.8%
Non-Black	622	53.1%
Age ^a^		
35 and under	577	53%
Over 35	512	47%

^a^ Counts do not add up to 1172 because of missing data on up to 7 percent of cases.

**Table 2:** Descriptive statistics and bivariate results for variables screened against parole decisions.

	Descriptive Statistic		Bivariate Relationship with Parole	
Variable	%	Mean (SD)	Chi-Square (χ^2^)/B Coefficient (SLR)	*p*
Parole Granted	58.5		---	---
**Control Variables: Parole Guidelines**				
LSI-R—total score		24.9 (9.74)	−0.64	***
Programming noncompliance	12.2		188.94	***
Successful program completion (any)	45.6		200.14	***
Violence prevention programing	23.5		90.61	***
Institutional misconduct	17.2		213.52	***
Misconduct, threat/blackmail	15.0		68.91	***
**Additional Control Variables**				
DOC/warden recommendation	60.8		219.00	***
Prior convictions (any)	60.0		3.09	+
Controlling offense—rape/sexual assault	8.7		76.20	***
Controlling offense—robbery	13.5		4.43	*
Controlling offense—property offense	18.0		5.96	*
Controlling offense—drug offense	25.2		17.31	***
Escape/walk-off attempt	28.2		7.06	**
Visitation	42.7		85.95	***
Age (over 35)	47.0		3.81	+
Medium custody level	38.9		9.01	**
Close custody level	19.7		58.65	***
Prior parole review/denial	31.2		23.92	***
Time served (in months)		48.4 (57.4)	-0.02	+
**Mental Health Variables**				
Self-reported mental health problems	11.6		1.98	+
LSI-R—Moderate interference	25.5		8.39	**
LSI-R—Severe interference, psychosis	5.1		2.89	+
LSI-R—Past mental health treatment	53.0		4.76	*
LSI-R—Present mental health treatment	30.4		7.86	**
LSI-R—Psych assessment indicated	20.1		19.49	***
PCRA—Anger management	24.1		33.29	***
PCRA—Impulsivity	38.5		23.74	***
PCRA—Suicide attempts	7.7		9.28	**
PCRA—Hallucinations/delusions	2.5		11.73	***
PCRA—Noncompliant with meds	4.4		11.86	***

Note. The parole decision was coded as 1 = recommended, 0 = not recommended. Results are summarized only from bivariate correlations with an associated significance level of *p* ≤ 0.25. We relied on crosstabs (*Chi-Squares* reported) for categorical variables, and simple logistic regression (*B coefficients* reported) for continuous variables. Relationships tested that did not meet this *p* ≤ 0.25 threshold are thus not shown here. + *p* ≤ 0.25, * *p* ≤ 0.05, ** *p* ≤ 0.01, *** *p* ≤ 0.001.

**Table 3:** Multiple logistic regression models predicting parole decisions: control variables, and self-reported, Level of Service Inventory-Revised (LSI-R), and Post-Conviction Risk Assessment (PCRA) mental health measures.

	Model 1		Model 2		Model 3		Model 4		Model 5	
Predictor	B (SE)	OR	B (SE)	B (SE)	OR	B (SE)	OR	OR	B (SE)	OR
LSI-R total score	−0.03 (0.01)	0.97 **	−0.04 (0.01)	−0.03 (0.01)	0.97 *	−0.02 (0.01)	0.98	0.97 **	−0.02 (0.01)	0.98
Programming noncompliance	−3.37 (0.53)	0.03 ***	−3.39 (0.53)	−3.53 (0.54)	0.03 ***	−3.49 (0.54)	0.03 ***	0.03 ***	−3.32 (0.53)	0.04 ***
Successful program completion (any)	1.15 (0.23)	3.17 ***	1.15 (0.23)	1.16 (0.23)	3.18 ***	1.23 (0.24)	3.41 ***	3.17 ***	1.21 (0.23)	3.36 ***
Violence prevention programming	1.04 (0.29)	2.83 ***	1.06 (0.30)	0.99 (0.30)	2.70 ***	0.97 (0.30)	2.62 ***	2.88 ***	0.98 (0.30)	2.67 ***
Institutional misconduct	−2.54 (0.32)	0.08 ***	−2.54 (0.32)	−2.50 (0.32)	0.08 ***	−2.49 (0.32)	0.08 ***	0.08 ***	−2.52 (0.32)	0.08 ***
Misconduct, threat/blackmail	−0.94 (0.29)	0.39 ***	−0.93 (0.29)	−0.92 (0.30)	0.40 **	−0.94 (0.30)	0.39 **	0.40 **	−0.96 (0.30)	0.38 ***
DOC/warden recommendation	0.97 (0.21)	2.63 ***	0.97 (0.21)	0.96 (0.21)	2.61 ***	0.99 (0.21)	2.69 ***	2.63 ***	1.00 (0.21)	2.71 ***
Prior convictions (any)	0.45 (0.19)	1.57 *	0.44 (0.19)	0.51 (0.19)	1.67 **	0.53 (0.20)	1.69 **	1.55 *	0.47 (0.19)	1.59 *
Controlling—Rape/Sexual Assault	−1.23 (0.38)	0.29 ***	−1.23 (0.38)	−1.28 (0.38)	0.28 **	−1.21 (0.39)	0.30 **	0.29 ***	−1.16 (0.39)	0.31 **
Controlling—Robbery	1.22 (0.33)	3.38 ***	1.22 (0.33)	1.28 (0.33)	3.61 ***	1.30 (0.34)	3.66 ***	3.38 ***	1.22 (0.33)	3.40 ***
Controlling—Property offense	0.89 (0.27)	2.43 ***	0.89 (0.27)	0.87 (0.27)	2.39 ***	0.87 (0.27)	2.39 ***	2.44 **	0.88 (0.27)	2.41 ***
Controlling—Drug offense	0.51 (0.24)	1.66 *	0.51 (0.24)	0.51 (0.25)	1.66 *	0.51 (0.25)	1.70 *	1.66 *	0.52 (0.25)	1.68 *
Escape/walk-off attempt	−0.50 (0.20)	0.61 *	−0.50 (0.20)	−0.52 (0.20)	0.60 *	−0.57 (0.21)	0.57 **	0.61 *	−0.54 (0.21)	0.58 **
Visitation	0.67 (0.20)	1.96 ***	0.67 (0.20)	0.67 (0.20)	1.95 ***	0.65 (0.20)	1.92 ***	1.96 ***	0.66 (0.20)	1.94 ***
Age (over 35)	−0.47 (0.22)	0.63 *	−0.47 (0.22)	−0.46 (0.22)	0.63 *	−0.47 (0.22)	0.63 *	0.63 *	−0.47 (0.22)	0.62 *
Medium Custody Level	−0.40 (0.23)	0.67	−0.41 (0.30)	−0.42 (0.23)	0.66	−0.51 (0.23)	0.60 *	0.67	−0.48 (0.23)	0.62 *
Close Custody Level	−0.64 (0.30)	0.53 *	−0.64 (0.30)	−0.67 (0.30)	0.51 *	−0.76 (0.31)	0.47 *	0.53 *	−0.73 (0.31)	0.48 *
Prior Parole Review/Denial	0.36 (0.23)	1.44	0.36 (0.23)	0.46 (0.24)	1.58	0.50 (0.24)	1.64 *	1.43	0.39 (0.23)	1.48 *
Time served (in months)	0.001 (0.002)	1.00	0.001 (0.002)	0.002 (0.002)	1.00	0.002 (0.002)	1.00	1.00	0.001 (0.002)	1.00
Self-Reported Mental Health	---	---	0.19 (0.29)	---	---	0.27 (0.33)	1.30	1.21	---	---
LSIR—Moderate Interference	---	---	---	---	---	0.13 (0.27)	1.14	---	0.11 (0.27)	1.12
LSIR—Severe Interference, Psychosis	---	---	---	---	---	0.29 (0.48)	1.34	---	0.17 (0.44)	1.18
LSIR—Past Mental Health Treatment	---	---	---	---	---	−0.10 (0.22)	0.90	---	−0.06 (0.22)	0.94
LSIR—Present Mental Health Treat	---	---	---	---	---	−0.33 (0.26)	0.72	---	−0.32 (0.26)	0.73
LSIR—Psych Assessment Indicated	---	---	---	---	---	−0.002 (0.28)	1.00	---	−0.02 (0.27)	0.98
PCRA—Anger Management	---	---	---	0.02 (0.27)	1.02	0.03 (0.27)	1.03	---	---	---
PCRA—Impulsivity	---	---	---	−0.50 (0.24)	0.61 *	−0.53 (0.24)	0.59	---	---	---
PCRA—Suicide Attempts	---	---	---	0.54 (0.38)	1.72	0.48 (0.39)	1.62	---	---	---
PCRA—Hallucinations/Delusions	---	---	---	−0.86 (0.62)	0.42	−0.95 (0.66)	0.39	---	---	---
PCRA—Noncompliant with Meds	---	---	---	0.10 (0.44)	1.11	0.13 (0.46)	1.14	---	---	---
**Constant**	0.27 (0.43)	---	0.26 (0.43)	0.30 (0.44)	---	0.06 (0.46)	---	---	0.04 (0.45)	---
***n***	1085		1085		1063		1085		1063	
**Chi-Square**	699.88		700.29		668.64		708.04		678.13	
**Df**	19		20		24		24		30	
**Pseudo (Nagelkerke) R^2^**	0.64		0.64		0.63		0.64		0.64	
**−2 Log Likelihood**	777.18		776.77		770.48		769.02		760.99	

Abbreviations: OR = odds ratios; SE = standard error. *** *p* ≤ 0.001; ** *p* ≤ 0.01; * *p* ≤ 0.05.
